# Reproductive history is associated with functional disabilities and symptoms in women with knee osteoarthritis: a case-control study

**DOI:** 10.1186/s13104-024-06859-9

**Published:** 2024-07-16

**Authors:** Fariba Eslamian, Seyed Kazem Shakouri, Narges Mohammadpour, Neda Dolatkhah, Soheila Bani, Fatemeh Nazari Khanmiri

**Affiliations:** 1 Physical Medicine and Rehabilitation Research Center, Tabriz University of Medical Sciences, Tabriz, Iran; 2 Obstetrics and Gynecology Surgery Resident, Faculty of Medicine, Tabriz University of Medical Sciences, Tabriz, Iran; 3 Gynecology Oncology Fellowship, Obstetrician-Gynaecologist, Shahriar Hospital, Tabriz, Iran

**Keywords:** Knee osteoarthritis, Reproductive history, Pain, Functional dysfunction

## Abstract

**Objectives:**

Studies that have examined the correlation between reproductive history and knee osteoarthritis (KOA) have had heterogeneous findings. We aimed to investigate the reproductive history and its relationship with pain and physical dysfunction in women with KOA. This case-control study, comprising 204 women aged 50 and older with and without KOA recruited through random cluster sampling, was executed from February 2018 to October 2018 in the health centers of Tabriz City. The reproductive history questionnaire was completed for the subjects in two groups. Pain intensity and functional dysfunction caused by KOA were evaluated using the Visual analogue scale and the Western Ontario and McMaster index, respectively.

**Results:**

The women’s age of menarche in the case group was significantly lower (*p* = 0.031), and the number of pregnancies (*p* = 0.017) and the average duration of breastfeeding (*p* = 0.039) were substantially higher than those of the control group. Older age at the first menstruation (OR = 0.851) was a protective factor, and higher parity (OR = 8.726) was a risk factor for KOA. In the women with KOA, the younger age of the mother at the birth of the first alive baby and the longer duration of breastfeeding were associated with higher pain intensity and functional disorders.

## Introduction

Osteoarthritis (OA) is the most common arthropathic disorder. Over 240 million world people are estimated to be diagnosed with OA [1]. OA causes pain and physical disabilities and decreases the elderly’s quality of life [2]. Because OA often develops in women in midlife, there has been ancient interest in the correlation between reproductive determinants and OA development and progression [3].

Scientific studies on the relationship between menarche [4] and menopause ages [4, 5], number of pregnancies leading to live births [5], history of hysterectomy [6], and radiographic or symptomatic OA show conflicting results.

For example, in a study of Korean women aged 50 or older, the number of pregnancies with healthy live births correlated positively with knee OA (KOA). However, no association was found between mode of delivery, use of birth control pills and hormone replacement therapy (HRT), duration of menstruation and breastfeeding after adjusting for age, body mass index (BMI), smoking, and education with KOA [7].

Individual heterogeneity of functional status in older people is well-recognized [8]. So the documentation of its elements may help recognize opportunities to prevent pain and functional disability. There is little inconsistent evidence regarding the correlation between endogenous sex hormones, reproductive factors, and hormonal supplements with OA, and much less in Iranian women with KOA. These studies are heterogeneous regarding study population characteristics, OA definition, and exposure measurement. In some countries, including Iran, KOA is more prevalent and develops at lower ages [9]. Therefore, considering the high prevalence of KOA in Iran, the heterogeneity of the Iranian populace, the lack of similar studies in Iran, and the emphasis of previous studies on the need to conduct more studies in this field, we decided to investigate the reproductive history of women aged 50 and older with KOA, and its correlation with pain intensity and functional dysfunction.

## Main text

### Method

#### Setting

This case-control study was conducted on the target population included all women aged 50 and higher diagnosed with KOA living in Tabriz.

The relative random sampling was done at the healthcare centers of Tabriz from February 2018 to October 2018. Tabriz has 82 healthcare centers in 10 different distinct areas. Six regions (2, 4, 5, 7, 8, and 9) were randomly selected, and one health center was chosen randomly from each. After that, the patients were contacted and invited to participate in a briefing meeting in a particular healthcare center. At the meeting, the study purposes and the methods that would have been used for doing the research were explained briefly, and the eligibility criteria were considered.

The inclusion criteria included women being diagnosed with primary KOA, based on the American College of Rheumatology criteria (primary OA with radiographic signs and symptoms of grade 1 to 3 of Kellgren and Lawrence) [10], having an age of 50 or above, using painkillers routinely (daily usage of acetaminophen or non-steroidal anti-inflammatory drugs in an edible or topical way), and having enough cognitive ability to fill the questionnaires.

The exclusion criteria included septic, neuropathic, and inflammatory arthritis, injecting corticosteroids in the past three months, consumption of opioids, using non-drug methods (physiotherapy) in the past six months, previous records of knee nerve blocking or recent knee injuries, joint replacement, reports of pathological fractures and degenerative radiographic arthritis in the lower limb joints except the knees, and inability to remember information that a relatively long time has passed, such as age at first menarche.

The participants in the control group were selected randomly from the same health center with a ratio of 1:1 after matching according to age and BMI. The participants in the control group had no symptoms of KOA and other arthropathic disorders, according to medical history and physical examination.

#### Procedure

The patient demographics, such as age, education, occupation, and history of chronic diseases, were collected using a general information questionnaire.

#### Reproductive history questionnaire

The reproductive history questionnaire included the age at the first menstruation, the woman’s age at the first pregnancy and childbirth, the number of pregnancies leading to the birth of live newborns, and the age at menopause [11]. The validity and reliability of the Persian version of the questionnaire were evaluated before being used. In this study, the Jones approach, which is a mixture of symmetrical and asymmetrical strategies, was used for translation [12]. In the first phase, two expert translators fluent in both English and Persian independently translated the original English questionnaire into Persian. The questionnaire was translated back into English. This version of the questionnaire was compared with the original version and it was shown that these two versions were almost alike. Finally, by translating this version into Persian, the final version of the questionnaire was attained. The final translation was given to the team of related experts to find the level of difficulty, incompatibility, ambiguity, or insufficiency in the meanings of the words.

The statistical technique of retesting and the correlation coefficient were applied to verify the reliability of the questionnaire.

The face validity of the questionnaire was checked by completing the questionnaire in the form of an interview with 20 women with KOA in the presence of the question designers. For this purpose, the Persian version of the questionnaire was examined in terms of fluency, clarity, and comprehensibility, as well as its appropriateness to the cultural conditions of our society. A 6-point scale was used (a score of 0 for completely vague, incomprehensible, and culturally inappropriate items to a score of 6 for completely expressive, understandable, and culturally appropriate ones). If 80% of women considered that question to have a score higher than 4, the question would be kept in the questionnaire. 85% of the respondents (*n* = 17) considered a score higher than 4 for every question.

To determine the content validity, the questionnaire was given to ten experts from the gynecology and midwifery department, and the scores of the content validity ratio (CVR) and the content validity index (CVI) were calculated.

The CVR and CVI for 28 questions of the questionnaire were obtained at an optimal level (CVI index for all questions was 0.82 and above and CVR for all questions was 0.80 and above).

The reliability of the questionnaire was checked by the test-retest method at an interval of 20 days. The correlation coefficient of the test-retest of the whole questionnaire was equal to *r* = 0.79 with a significance of *p* = 0.005. Thus, in more than 75% of all the questions, the respondents gave the same answers in two times of the questionnaire.

#### Visual analogue scale (VAS)

The pain intensity was measured by the Visual Analogue Scale (VAS). VAS is one of the most frequently used tools for evaluating pain intensity [13].

#### Western Ontario and McMaster Index (WOMAC)

The Western Ontario and McMaster index (WOMAC) is one of the most valuable tools for measuring the functional status and pain caused by OA in the lower limb [14]. We used the Persian version with proven validity and reliability [15].

### Statistical analysis

To determine the sample size, the relationship between the number of pregnancies and pain in OA patients was considered the main objective, and the primary data was the average correlation coefficient based on the Cochrane criterion (*r* = 0.3). With a 5% error type 1, 80% power, two-tailed test, and using the formula:

$$n = \frac{{{{\left( {{Z_{1 - \frac{\alpha }{2}}}{Z_{1 - \beta }}} \right)}^2}}}{{{{\left( \omega \right)}^2}}} + 3$$

Wherein:

$$\omega = \frac{1}{2}Ln\frac{{1 + r}}{{1 - r}}$$

the minimum sample size for both groups was calculated as 85 cases. Calculating an incomplete data of 20%, the final sample size was increased to 102.

The chi-square test and the independent t-test (Mann-Whitney, if the assumptions of the independent t-test are not established) were used to compare the qualitative and quantitative data, respectively. Spearman’s correlation coefficient was used to check the relationship between quantitative variables. Logistic regression analyses were applied to calculate the odds ratio (OR) for the outcome (KOA) with independent variables (age at menarche, age at menopause, number of pregnancies, duration of use of hormone replacement drugs, number of children and pregnancies, and total duration of breastfeeding). The significance level in all tests was considered 0.05. SPSS version 16 software was used for data analysis.

## Results

We assessed 247 women with KOA for eligibility, from which 154 were eligible to participate. Finally, 102 women with KOA agreed to participate and were recruited as the case group. One hundred and thirty-one healthy women were assessed for eligibility, from which 102 women were eligible and agreed to participate and recruited as the control group. The demographics and medical history information of the participants are shown in Table 1. No significant differences were observed demographically and by the medical history between the control and case groups except for the intake of supplements.

**Table 1:** Demographic and clinical characteristics of the participants

Variable		Case (*n* = 102)	Control (*n* = 102)	*P*
Age, (mean ± SD) (Yrs)		56.96 ± 6.68	56.72 ± 4.80	-
Age, *n* (%) (Yrs.)	51–60	71 (69.6)	71 (69.6)	-
	61–70	31 (30.4)	31 (30.4)	
Education, *n* (%)	Illiterate	35 (34.3)	27 (26.5)	0.158*
	Secondary School	49 (48.0)	57 (55.9)	
	Diploma	4 (4.0)	10 (9.8)	
	University	14 (13.7)	18 (7.8)	
Job, *n* (%)	Housewife	75 (73.3)	82 (80.4)	0.244*
	Employed	6 (5.9)	7 (6.9)	
	retired	21 (20.8)	13 (12.7)	
Kellgren and Lawrence grade of radiographic severity (most affected), n (%)	I	31 (30.4)		-
	II	41 (40.2)		
	III	30 (29.4)		
Weight (Kg), Mean ± SD		78.21 ± 9.89	79.95 ± 13.43	0.881**
Height (cm), Mean ± SD		162.82 ± 5.77	161.76 ± 5.47	0.697**
BMI (kg/m2), Mean ± SD		29.53 ± 3.73	30.60 ± 5.19	-
BMI (kg/m2), *n* (%)	18.5≥	6 (6.0)	6 (6.0)	
	18.5–29.4	33 (32.3)	33 (32.3)	
	25≤	63 (61.7)	63 (61.7)	
Concurrent chronic disease, *n* (%)	Yes	53 (52.0)	61 (52.8)	0.508*
	No	49 (48.0)	41 (47.2)	
Medication use for concurrent chronic disease, *n* (%)	Yes	37 (36.3)	47 (46.1)	0.637*
	No	65 (63.7)	55 (53.9)	
Food Supplement intakes, *n* (%)	Yes	51 (50.0)	26 (25.5)	0.003*
	No	51 (50.0)	76 (74.5)	

*chi-square test

**Independent t-test

The pain intensity (VAS) was 5.65 ± 1.28, and the physical dysfunction (WOMAC total) was 64.63 ± 8.08 in the participants in the case group. The WOMAC pain, WOMAC stiffness and WOMAC physical dysfunction scores were 11.83 ± 1.82, 3.77 ± 1.16 and 48.93 ± 6.43 points, respectively.

The reproductive history of the participants is shown in Table 2. The correlations of the reproductive history variables with the WOMAC and VAS indices are shown in Table 3. There was a statistically significant negative correlation between the age at menarche and pain intensity according to the WOMAC pain score (*r*=-0.222, *p* = 0.028). There were statistically significant negative correlations between the age at the time of the first live birth with physical dysfunction according to the WOMAC total score (*r*=-0.237, *p* = 0.021), and WOMAC physical function (*r*=-0.209, *p* = 0.040), and pain intensity according to WOMAC pain score (*r*=-0.258, *p* = 0.011) and VAS (*r*=-0.202, *p* = 0.048). There was a statistically significant positive correlation between the total duration of breastfeeding with physical dysfunction according to the WOMAC physical function score (*r* = 0.213, *p* = 0.047), and with pain intensity according to the WOMAC pain score (0.261 = *r* = 0.261, *p* = 0.014) and VAS (0.279 = *r* = 0.279, *p* = 0.009).

**Table 2:** Reproductive history of the participants

Variable		Case (*n* = 102)	Control (*n* = 102)	*P*
Menarche Age, (mean ± SD) (Yrs)		10.46 ± 1.02	13.36 ± 1.26	0.031
Menopause, *n* (%)	Natural	77 (75.5)	87 (85.3)	0.267*
	Surgery	25 (24.5)	11 (14.7)	
Menopause Age, (mean ± SD) (Yrs)		48.04 ± 7.48	50.24 ± 4.47	0.071
HRT, *n* (%)		5 (4.9)	16 (15.6)	0.518*
HRT Type, *n* (%)	Megestrol acetate	5 (100.0)	12 (75.0%)	0.746*
	Medroxyprogesterone	0 (0.0)	4 (25.0%)	
Duration of use of hormone replacement drugs, (mean ± SD) (months)		10.28 ± 4.99	9.00 ± 4.24	0.017***
Number of live births, *n* (%)	1–3	52 (51.0)	86 (84.3%)	0.015*
	4≥	50 (49.0)	16 (15.7%)	
Number of abortions, *n* (%)	0–1	89 (87.3)	100 (98.1%)	0.008*
	2–3	13 (12.8)	2 (1.9%)	
Mother’s age at the time of the first live birth, (mean ± SD) (Yrs)		17.47 ± 2.85	19.29 ± 3.33	0.208**
Maternal age at first pregnancy, (mean ± SD) (Yrs)		16.55 ± 3.08	18.33 ± 5.92	0.311**
Breastfeeding history, *n* (%)		88 (87.1)	83 (81.4)	0.262*
Total duration of breastfeeding, (mean ± SD) (months)		16.52 ± 9.98	10.32 ± 7.76	0.039***
Ovariectomy history, *n* (%)	One-sided	8 (80.0)	4 (100.0%)	0.447*
	Two-sided	2 (20.0)	0 (0.0%)	
Age at time of ovariectomy, (mean ± SD) (Yrs)		38.27 ± 11.64	37.00 ± 3.46	0.669**
History of hysterectomy, *n* (%)		25 (24.5)	11 (14.7)	0.267*
Age at hysterectomy, (mean ± SD) (Yrs)		37.79 ± 5.49	44.00 ± 4.64	0.021**
History of infertility, *n* (%)		0 (0.0)	3 (2.5)	0.851*
History of using assisted reproductive methods, *n* (%)		6 (5.8)	3 (2.5%)	0.697*
Use of contraceptive methods, *n* (%)	Birth control pills	2 (20.0)	0 (0.0%)	0.269*
	Long-term progesterone injection	44 (43.1)	39 (38.2%)	
	intrauterine device (IUD)	6 (5.8)	12 (11.8%)	
	Tubectomy	7 (6.9)	10 (9.8%)	
	Vasectomy	0 (0.0)	10 (9.8%)	
	Condom	4 (3.9)	7 (6.8%)	
Duration of contraceptive method use (months)	Birth control pills	25.13 ± 21.46	20.00 ± 13.04	0.187***
	Long-term progesterone injection	25.23 ± 30.20	31.09 ± 13.68	0.094***
	intrauterine device (IUD)	44.73 ± 35.85	42.27 ± 15.32	0.335***
	Condom	31.20 ± 10.73	36.00 ± 00.00	0.164***
History of breast cancer screening, *n* (%)		15 (14.7)	18 (17.6)	0.569*

*chi-square test

**Independent t-test

*** Mann-Whitney U test

Note: A *p*-value less than 0.05 is considered a statistical significance level. Note:

**Table 3:** Correlation of reproductive history with pain intensity and functional dysfunction in knee osteoarthritis

	WOMAC total	WOMAC physical function	WOMAC stiffness	WOMAC Pain	VAS
**Menarche Age**	0.165-= r 0.107 = *p*-value	0.127-= r 0.213 = *p*-value	0.076-= r 0.456 = p-value	0.222-= r 0.028 = *p*-value	0.015-= r 0.886 = *p*-value
**Menopause Age**	0.181 = r 0.118 = *p*-value	0.147 = r 0.147 = *p*-value	0.191 = r 0.059 = *p*-value	0.137 = r 0.179 = *p*-value	0.044 = r 0.670 = *p*-value
**Age at the time of the first live birth**	0.237-= r 0.021 = *p*-value	0.209-= r 0.040 = *p*-value	0.013-= r 0.913 = *p*-value	0.258-= r 0.011 = *p*-value	0.202-= r 0.048 = *p*-value
**Age at first pregnancy**	0.185-= r 0.071 = *p*-value	0.165-= r 0.106 = *p*-value	0.036-= r 0.728 = *p*-value	0.188-= r 0.064 = *p*-value	0.095-= r 0.353 = *p*-value
**Total duration of breastfeeding**	0.185 = r 0.102 = *p*-value	0.213 = r 0.047 = *p*-value	0.148-= r 0.166 = *p*-value	0.261 = r 0.014 = *p*-value	0.279 = r 0.009 = *p*-value

Spearman’s correlation coefficient analysis was used. *P* value less than 0.05 is considered as statistical significance level

VAS: Visual analogue scale; WOMAC: Western Ontario and McMaster

For multivariable analyses of reproductive history variables and KOA, we fit univariate and multivariable models with the following variables as independent variables: age at menarche, age at menopause, number of pregnancies, duration of use of hormone replacement drugs, number of children and pregnancies, and total duration of breastfeeding. According to logistic regression results, a higher age at menarche (OR = 0.851, 95% CI: 0.753–0.962) was a protective factor, and a higher number of pregnancies (OR = 8.726, 95% CI: 3.498–21.764) was a risk factor for KOA in the investigated women.

## Discussion

According to our findings, patients with KOA had significantly younger ages at menarche and hysterectomy compared to matched controls without KOA. However, patients with KOA had a higher duration of use of HRT, a higher number of live births and abortions, and a longer duration of breastfeeding compared to match controls without KOA. Younger age at menarche and at the time of the first live birth correlated with higher pain intensity measured by WOMAC pain score. Younger age at the time of the first live birth correlated with higher pain intensity measured by VAS. Furthermore, longer duration of breastfeeding positively correlated with physical dysfunction according to WOMAC physical function and pain intensity according to WOMAC pain score and VAS in patients with KOA. A higher age at menarche was a protective factor, and a higher number of pregnancies was a risk factor for KOA. To our knowledge, this is the first study that investigated comprehensively the reproductive history and its correlation with pain and disability in Iranian women with KOA.

Earlier cross-sectional and case-control investigations failed to detect any correlation between parity and KOA [16–18]. However, a large cohort study including more than one million women from the United Kingdom has revealed positive correlations between younger age at menarche and increasing parity with total knee replacement (TKR) incidence [4]. A cohort study established an elevated risk of OA with a higher number of offspring in both men and women, with a most notable correlation for KOA in women [19]. A recent investigation from Norway stated that the risk of TKR reduced with older age at menarche with no correlation with parity [5].

In line with our findings, some authors have proposed the probability that younger age at menarche correlates with an elevated rate of the general aging process and other aging situations such as high blood pressure and glucose intolerance [20]. Canonico et al. [21] indicated that a younger age at menopause and a higher number of births have a detrimental effect on the physical function that persists in older people in a cohort study on 3043 French women. Investigating the role of the age of first menarche on the probability of OA in different studies has shown different results, which can be due to various causes, including the difference in the method of obtaining the age of menarche, which in the present study is asked from the patient himself, which can be subjected to various types of bias. The mechanisms underlying these correlations are unclear. One possible justification could be that younger age at menarche may be an indicator of higher BMI when young [4] which is an important risk factor for OA later in life [22]. A cross-sectional investigation found an association between younger age at menarche and chronic general musculoskeletal disorders later in life [23]. Younger age at menarche has also been associated with other aging disorders such as high blood pressure and insulin resistance, independent of body composition [20]. Some previous investigations revealed younger age at menarche [24, 25] and longer menstrual period [24] to be independent risk factors for hand OA, which is less predisposed by body weight. This finding is exciting as it proposes that a younger age of exposure or a longer exposure to cyclic hormonal changes throughout the menstrual cycle because of a younger age at menarche may have unfavorable effects on the knees.

Endogenous sex hormones may have unique effects on the lifetime risk of OA by preserving articular cartilage and subchondral bone [26]. It has been shown that sex steroids have multifaceted effects on articular cartilage through direct actions on chondrocytes and are involved in OA-associated alterations, but many details remain to be discovered [27]. Changes in sex hormones may affect bone turnover through estrogen receptors (ERs) in the musculoskeletal system and disturb the metabolic balance of bone and cartilage [28]. The investigators proposed that the hormonal effects may result from estrogen’s effects on opioid pathways or direct effects on bone and cartilage, as other studies have reported that women who received estrogen or alendronate had less OA-related subchondral bone wear and bone marrow edema abnormalities than women who did not receive these drugs [29]. However, dissimilarities between correlations at different joints in different populations, complexities of observational versus trial documents, and lack of recent documents regarding reproductive history and joint health in our country (Iran) make comparisons and inferences problematic in practice.

Previous investigations on the correlation between parity and KOA have shown contradictory results [4, 6, 30]. The combination of a considerable transient weight gain and increased joint laxity in pregnancy might explain, at least in part, the findings of an increased long-term risk of OA of the knee in women with multiple offspring. Alternatively, a possible association between childbirth and subsequent risk of OA of the knee might also be related to the estrogen and progesterone receptors in cartilage that might be affected by the elevated levels of sex hormones during pregnancy [31]. Parity is independently related to lower cartilage volume, principally in the tibial section, and higher cartilage defects in the patella section in women aged over 50 years [6]. Reproductive and hormonal factors are also associated with obesity. In addition, some women gain weight during menstruation and pregnancy. Obesity and weight gain are definite risk factors for OA [32]. Endogenous hormones and reproductive factors through obesity may create an inflammatory environment and consequently promote the growth and progress of OA. On the other hand, low levels of estradiol can create an inflammatory environment in the joint by inducing inflammatory stressors through the anti-inflammatory properties of ERb [33].

In line with our findings, Eun et al. [34] suggested that prolonged breastfeeding and use of HRT and contraceptives were associated with an increased risk of total knee replacement due to severe OA in a cohort study, even though older age at menopause and Longer reproductive age were associated with reduced risk. Estrogen deficiency, through menopause or hysterectomy, is recognized to be related to OA [35], but the exact mechanism is uncertain. The prevalence and severity of OA are greater in postmenopausal women than in premenopausal women [36]. Lactation is another physiological period of low estrogen situation [37]. The positive correlation between breastfeeding and OA shows the necessity to provide prevention knowledge, especially for women who breastfeed.

Several epidemiologic instantiations have studied the correlation between OA [38] or joint replacement [5] and exogenous hormonal factors such as oral contraceptives [38] and HRT [39]. However, the findings of these investigations are often inconsistent. This inconsistency might be described by indication bias: HRT is prescribed for women with estrogen deficiency symptoms. So, it can be considered an index of estrogen lack rather than a consequence of additional estrogen. The type of hormone supplements may also be essential. In the Cirrillo et al. study [40], Caucasian women who received estrogen alone were less likely to undergo arthroplasty, especially in the hip area (less in the knee area), and women who received combined hormone therapy had less pain and joint stiffness.

## Conclusion

In this study, we found that younger ages at menarche and hysterectomy, longer use of HRT, a higher number of live births and abortions, and longer duration of breastfeeding were associated with a higher risk of KOA. KOA women with younger age at menarche and at the time of the first live birth and longer duration of breastfeeding experienced higher pain intensity. Furthermore, KOA women with longer duration of breastfeeding experienced higher physical dysfunction. There are practical concepts in our study. Younger age at menarche and higher numbers of pregnancies can increase the risk of KOA. So, clinicians should pay attention to adolescents who experience early menarche and young women who experience several pregnancies to ensure that they obtain proper preventive management. This is while the recent policies of Iran are currently based on having children at a younger age and more often, and this issue is critical in preventing OA in these women in the future. In addition, lifestyle modifications should be considered for women with longer use of HRT, a higher number of live births and abortions, and a longer duration of breastfeeding to prevent early KOA and following joint replacement therapy.

### Limitations

The observational and retrospective nature of the study, subjective reproductive data (self-reported), not determining the blood estrogen concentration, and the relationship between hormone-related factors and KOA were the limitations.

## Data Availability

All the necessary data are presented herewith. However, if needed, raw data on excel format can be availed on reasonable request from the corresponding author.

## Abbreviations

BMI: Body mass index
CVI: Content validity index
CVR: Content validity ratio
ER: Estrogen receptor
HRT: Hormone replacement therapy
KOA: Knee osteoarthritis
OA: Osteoarthritis
VAS: Visual Analogue Scale
WOMAC: Western Ontario and McMaster Index
